# Intra-articular platelet-rich plasma injections versus intra-articular corticosteroid injections for symptomatic management of knee osteoarthritis: systematic review and meta-analysis

**DOI:** 10.1186/s12891-021-04308-3

**Published:** 2021-06-16

**Authors:** Michael McLarnon, Neil Heron

**Affiliations:** 1 Ballymena, UK; 2 School of Medicine, Dentistry and Biomedical Science, Queens University Belfast, Belfast, United Kingdom; 3 Centre for Public Health Research, Queen’s University, Belfast, UK; 4 UKCRC Centre of Excellence for Public Health Research (Northern Ireland), Belfast, United Kingdom; 5 Department of General Practice, Keele University, Newcastle, England

**Keywords:** Systematic review, Meta-analysis, Knee, Osteoarthritis, Platelet-rich plasma, Corticosteroids, Intra-articular

## Abstract

**Background:**

Intra-articular (IA) corticosteroid (CS) injections are the mainstay of treatment for symptomatic management in knee osteoarthritis (OA), particularly in the UK. IA platelet-rich plasma (PRP) injections are a promising alternative, but no systematic reviews to date have compared them to the current standard of care, IA CS injections. We aim to investigate the effect of IA PRP injections versus IA corticosteroid injections for the symptomatic management of knee OA.

**Methods:**

All published trials comparing IA PRP and CS injections for knee OA were included. MEDLINE, EMBASE, Scopus and Web of Science were searched through June 2020. Risk of bias was assessed using the Cochrane Risk of Bias tool. A random effects model was used to calculate standardized mean difference with 95% confidence interval in WOMAC/VAS score (or subscores), comparing IA PRP to CS injections across studies.

**Results:**

Included were eight studies and 648 patients, 443 (68%) were female, mean age 59 years, with a mean BMI of 28.4. Overall, the studies were considered at low risk of bias. Compared with CS injections, PRP was significantly better in reducing OA symptoms (pain, stiffness, functionality) at 3, 6 and 9 months post-intervention (*P < 0.01*). The greatest effect was observed at 6 and 9 months (− 0.78 (− 1.34 to − 0.23) standard mean deviations (SMD) and − 1.63 (− 2.14 to − 1.12) SMD respectively). At 6 months, this equates to an additional reduction of 9.51 in WOMAC or 0.97 on the VAS pain scales. At 6 months PRP allowed greater return to sporting activities than CS, measured by the KOOS subscale for sporting activity, of magnitude 9.7 (− 0.45 to 19.85) (*P = 0.06*). Triple injections of PRP, generally separated by a week, were superior to single injections over 12 months follow-up (*P < 0.01*).

**Conclusions:**

IA-PRP injections produce superior outcomes when compared with CS injections for symptomatic management of knee OA, including improved pain management, less joint stiffness and better participation in exercise/sporting activity at 12 months follow-up. Giving three IA-PRP, with injections separated by a week, appears more effective than 1 IA-PRP injection.

**Prospero trial registration number:**

CRD42020181928.

**Supplementary Information:**

The online version contains supplementary material available at 10.1186/s12891-021-04308-3.

## Background

### Prevalence and impact of knee osteoarthritis

Musculoskeletal (MSK) conditions make up a significant workload in general practice (GP) and musculoskeletal services, with one in seven GP consultations being for MSK conditions [1]. Patients frequently present to their GP with knee osteoarthritis (OA) symptoms [2] and a recent analysis of primary care musculoskeletal referrals to secondary care [3] found that knee OA was the commonest reason for orthopaedic secondary care referral. Moreover, symptomatic OA is one of the leading causes of adult disability in the world, with significant economic impact [4]. Adding to this, the burden of knee OA continues to grow [5], estimated to affect between 10 and 25% of patients over 60 [4], alongside a population that is increasingly co-morbid and obese [5, 6].

### Current treatment paradigm

The United Kingdom (UK) has national guidelines for the management of OA [7]. These guidelines include the use of intra-articular (IA) corticosteroid (CS) and local anaesthetic injections, followed by exercise prescription [7]. Only a small proportion of patients with knee OA meet physical activity guidelines due to the impact of pain on their functioning [8]. Such findings place even greater emphasis on the necessity for effective pain management options, including IA injections, especially in a climate with a lengthy waiting time for surgical intervention [9, 10], which negatively impacts patient quality of life [11].

IA CS injections are often prescribed before secondary care referral, attempting to provide symptomatic management and delay surgery. Although CS injections appear to improve pain scores in osteoarthritic patients for a limited time period [12], they are associated with side-effects [13] and do not appear to offer symptomatic improvement for longer than 6 weeks [12]. Indeed, some authors [13] have advised against using IA CS therapy because of the deleterious effects on articular cartilage [14], leading to a deterioration of the underlying joint OA. Previous studies have shown a statistically significant additional deterioration in articular cartilage compared to placebo, as well as an increased propensity for knee replacement in patients treated with CS injections [13, 15].

During the current COVID-19 pandemic of 2020, CS injections are being used judiciously due to the impact of CS on immunity and alternatives must be considered. Thus, research is needed to identify and show the effects of new management options for patients with knee OA, particularly to offer better non-operative pain management.

### Platelet-rich plasma as an emerging therapy

One such option might be IA platelet-rich plasma (PRP) injections, shown to reduce pain and improve function for knee OA patients in systematic reviews and meta-analyses [16, 17], with improvements reported to last up to 1 year [16]. IA PRP is not associated with the deleterious effects on cartilage that IA CS use is, making it a safer option, particularly when repeated injections may be required. IA PRP injections have been shown to have positive effects on chondrogenesis and mesenchymal stem cell proliferation [17, 18]. IA PRP injections have also been shown to decrease inflammatory markers and promote anti-inflammatory mediators, as well as reducing the expression of inflammatory enzymes [17, 19]. Therefore, there is strong biological plausibility to advocate IA PRP injections as a potential alternative to CS injections. However, IA PRP is not currently offered as a standard treatment to those with knee OA, and most studies do not compare IA PRP to IA CS/local anaesthetic injections; the current standard of care in the UK. There is also some debate surrounding how many times IA PRP injections should be given, with some authors suggesting using 3 injections [20, 21], separated by a week, whilst others use only one injection [22]. .PRP injections can also contain different concentrations of leukocytes and there is discourse regarding which is superior, leukoocyte-rich PRP (LR-PRP) as opposed to leukoocyte-poor PRP (LP-PRP) [23]. Thus a systematic review of the evidence is required to compare IA PRP to IA CS injections for the symptomatic management of knee OA, as well as to consider the effectiveness of the different forms of PRP (LR-PRP versus LP-PRP) and the number of IA PRP injections to be given as a course of treatment.

### Aim

The primary aim is to investigate the effect of IA PRP injections versus IA CS injections for the symptomatic management of adults with knee OA. Secondary aims are to identify the effectiveness of one IA PRP injection compared to multiple injections for one course of treatment and whether LR-PRP is more effective to LP-PRP injections in the management of knee OA.

## Methods

This review is reported as per the Preferred Reporting Items for Systematic Reviews and Meta-analyses (PRISMA) guidelines [24] and the review was prospectively registered in PROSPERO (registration number CRD42020181928). Methods of the analysis and inclusion criteria were specified in advance and documented in a protocol (submitted for publication).

### Search methods for identification of studies

Detailed search strategies were developed for each electronic database searched, with input from a medical librarian. These have been based on the search strategy developed for Medical Literature Analysis and Retrieval System Online (MEDLINE) but were revised appropriately for the following databases: EMBASE, Scopus and Web of Science. An example of our search strategy is included as a supplementary file (Additional file 1). No limits were applied for language or timeframe. The most recent search was performed in June 2020. Any studies directly comparing injectable PRP with injectable corticosteroids for the management of symptomatic knee osteoarthritis were screened by both authors for additional references. The reviewers supplemented the electronic search strategy by using the Science Citation Index to perform citation tracking of the trials identified by the first step.

### Study inclusion and exclusion criteria

Inclusion criteria: Adults (Aged 18 or over) with symptomatic knee OA receiving IA PRP injections for symptomatic management of knee OA and comparing this treatment to IA CS injections.

Exclusion criteria: Adolescents (under 18 years of age); studies using co-interventions alongside either PRP or CS and studies not comparing IA PRP directly to IA CS.

### Types of outcome measures

#### Primary outcome

- Change in score using the Western Ontario and McMaster Universities Osteoarthritis Index (WOMAC) and Knee Injury and Osteoarthritis Outcome Score (KOOS) scores as well as the visual analog scales (VAS) at 6 months post-intervention.

#### Secondary outcomes

- Outcomes at 1, 3, 9- and 12-months post-intervention were assessed where available.
- Stratification of analyses by Kellgren Lawrence (KL) grade were performed where possible.
- Individual sub-scores as well as overall score from each scoring tool is assessed.
- Efficacy of single versus multiple injections of platelet-rich plasma.

### Data collection and analysis

#### Selection of studies

The titles and abstracts of publications obtained by the search strategy were independently screened by one author (MM). Articles that did not meet the inclusion criteria were removed. All remaining publications were retrieved for further assessment by two independent authors (MM and NH). Based on the information within the full reports, the final articles were then selected for inclusion in the review. A record was kept of all articles excluded at this stage and the reason for their exclusion. Each included trial was required to contain extractable data for at least one of the outcome measures of pain commonly recommended for the assessment of knee OA (WOMAC, KOOS and/or VAS).

### Data extraction and management

Two reviewers (MM,NH) applied pre-agreed eligibility criteria to select studies for inclusion. One researcher screened (MM) and the senior author (NH) reviewed decisions, with researchers being blinded to the other’s decisions. Disagreements were resolved through discussion of the study’s methodology and included/removed at this point. Relevant data was extracted on prospective trial methodology; study participants (including grade of knee OA); study length (including year initiated); country of origin/nationality of participants; eligibility criteria; interventions (including the number of injections received, dosage of injection, method of delivery and type of PRP used (LR-PRP or LP-PRP)) and study design; study duration; follow-up; comparisons; outcome measures and results. Attempts were made where necessary to contact original investigators to request missing data. There was no blinding to study author, institution or journal.

### Assessment of risk Bias and heterogeneity

One review author (MM) independently assessed each included study for risk of bias using the Risk of Bias 2 tool [18], following guidance from the Cochrane Handbook of Systematic Reviews of Interventions [19], with decisions reviewed by the senior author (NH). At this point, articles were also assessed for clinical and methodological heterogeneity.

### Measures of treatment effect

The difference in mean, with corresponding standard error (SE), was extracted from each study as measured by VAS, WOMAC and KOOS at different time points of follow-up for symptomatic management of knee OA. Where studies used different outcome measures, the reviewers combined the relevant studies using the same outcome measure.

### Data synthesis

A random effects model was used to calculate a pooled standardised mean difference (MD) with 95% confidence intervals (CI) in WOMAC/VAS score (or subscores) comparing IA PRP to CS injections across studies. A SMD was used as the WOMAC/VAS measure the same underlying construct, pain. This analysis was repeated calculating the pooled mean difference (MD) restricted to studies that reported the same pain outcome. In one study, the median and range was converted to a mean and standard deviation using formulae described by Hozo et al. [25]. A random-effects model was used in all pooled analyses as we anticipated clinical heterogeneity. The Chi-squared test for heterogeneity was conducted and I^2^ value was used to measure heterogeneity. Publication bias was assessed from inspection of funnel plots (an example from our primary analysis is included as Additional file 2). All analysis was performed using Review Manager 5.4.

## Results

### Search results

**Fig. 1:**
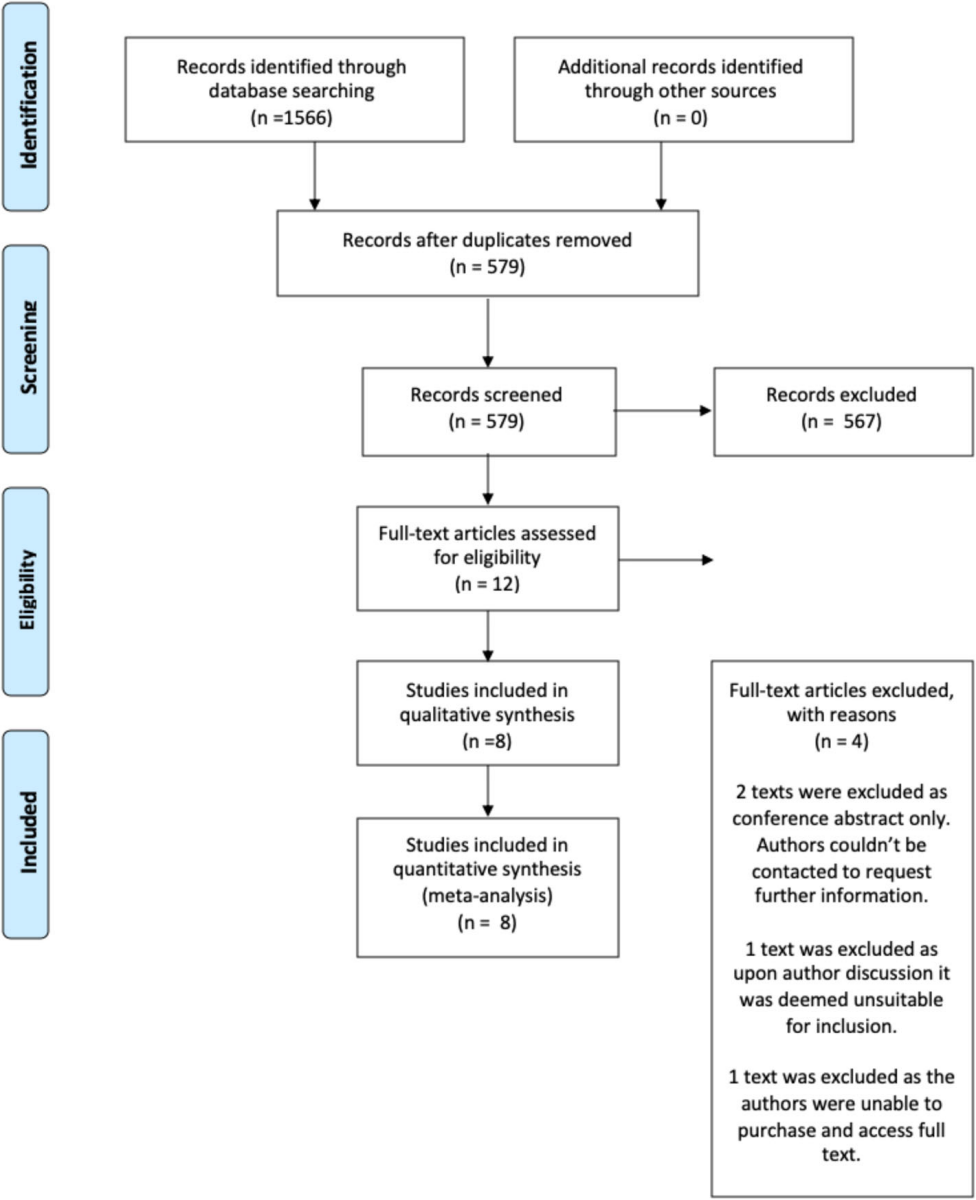
Summary of the Search Results and Trial Selection

Our search yielded 1566 studies, of which there were 579 original articles after duplicates were removed. Titles and abstracts were screened for 579 articles, at which point 567 were excluded as irrelevant to the study question. Full texts were identified for the remaining 12 texts. In total, eight trials were included within the analysis, published between 2017 and 2019. Details of the literature search are demonstrated in the flow chart (Fig. 1).

### Study characteristics

The characteristics of the eight included trials are presented in Table 1. The studies included 648 adult patients, with 206 (32%) being male. Mean patient age was 59 years. Radiographically, all studies used the Kellgren-Lawrence (KL) grading systems to determine severity of knee OA, with pooled information for six studies showing that 0.2% were grade I, 45% were grade II, 45% were grade III, and 9% were grade IV. Huang et al. [26] and Phul et al. [27] only reported average KL grades for CS and PRP groups (KL 1–2 and 2–4 respectively) and therefore were not included in the grade-percentage stratification above. The mean BMI of patients was 28.4 across six studies, with Freire et al. [28] simply reporting that 82% of their patients were obese and Phul et al. [27] that all patients had a BMI < 33 kg/m^2^.

**Table 1:** Summary of Trial Characteristics

Study	Sample (n)	Dropouts	Study Design	Population	Intervention design	Follow-up period	Outcome Measurement
Huang et al. (2019) [26]	265 recruited 120 participants (46% female) 40 received PRP, 40 received CS and 40 HA (HA patients not included)	0 145 did not meet inclusion criteria	Prospective, randomised study	Kellgren/Lawrence grade 1–2 Aged 40–65 Chinese patients recruited from May 2016	1 injection per group, received once Anatomically guided injections 4 ml LP-PRP 1 ml corticosteroid	12 months	WOMAC and VAS scores at 3, 6,9- and 12-months post intervention
Khan et al. (2018) [29]	150 patients recruited 101 participants (76.5% female) 52 received PRP, 51 received CS	49	Randomised control trial	Kellgren/Lawrence grade 2 and ACR osteoarthritis criteria Aged 40–63 Pakistani patients recruited between July ‘17-July ‘18	2 injections per group, given at 0 months and 2 months. Anatomically guided injections 5 ml PRP (LR/LP not specified) 1 mil (40 mg) triamcinolone	6 months	WOMAC and VAS scores at 2 months, then every 2 weeks until 6 months
Naderi et al. (2018) [30]	72 recruited 67 participated (82% female) 33 received PRP, 34 received corticosteroids	5	Randomised control trial with Quad ruple block randomisation	Kellgren/Lawrence or ACR grade 2–3 osteoarthritis Aged 30–75 Iranian patients between April ‘16- June ‘17, recruited in a hospital	3 injections performed once a month for 3 consecutive months in both groups Ultrasound guided injections 5 ml LR-PRP 40 mg triamcinolone	6 months	VAS and KOOS scores before the onset of treatment, once a month after treatment and 6 months after treatment.
Freire et al. (2018) [28]	50 patients (25 PRP, 25 corticosteroids) (84% female) Double-blinded block randomisation	0	Randomised, controlled, longitudinal, double blind, comparative, descriptive study	Kellgren/Lawrence 2–4 Aged 30–90 Brazilian patients, recruited in a medical centre	1 injection performed once in each group Anatomically guided injections 5 ml LP- PRP 2.5 ml triamcinolone	6 months	KSS, WOMAC, Kellgren-Lawrence scores evaluated before treatment, at 1 months and 6 months post-intervention
Camurcu et al. (2018) [31]	132 patients recruited (83% female) 37 PRP, 40 PRP+ steroid, 38 steroid	17	Prospective longitudinal study	Kellgren-Lawrence grade 2–3 Aged 40–80 Turkish patients recruited in 2016	1 injection of PRP only, PRP + steroid, or steroid only Anatomically guided injection 3 ml LR-PRP 1 ml methylprednisolone	12 months (for 115 patients)	VAS and WOMAC at 1,3, 6- and 12-months post-intervention
Phul et al. (2018) [27]	80 patients recruited 40 in each group: PRP or corticosteroid (67.5% female)	0	Randomised control trial	Kellgren/Lawrence grade 2–4 Aged 40–75 Pakistani patients recruited between Feb- August 2015	1 injection in both groups Anatomically guided injection for PRP; fluoroscopically guided corticosteroid injection 4–6 microml LR-PRP 40 ml triamcinolone	3 months	VAS at baseline and 3 months post-intervention
Uslu et al. (2017) [20]	57 patients recruited 50 participants (92% female) 17 received CS, 19 single PRP, 14 triple PRP injection	7	Prospective, randomised, single-blind (physician blind)	Kellgren/Lawrence grade 3 Aged 50–75 Turkish patients between June ‘15- March ‘16, hospital setting	3 groups receiving either 1 PRP injection, 3 PRP injections (given 1 week apart) or 1 steroid injection Anatomically guided injections LR-PRP, dose not specified Corticosteroid suspension equivalent to 5 mg betamethasone	6 months	VAS, WOMAC and HAD scale (an assessment of anxiety and depression) with follow up at baseline, 2 and 6 months post-intervention
Jubert et al. (2017) [22]	65 patients included (72% female) 35 PRP, 30 corticosteroid	0	Prospective randomised, double-blind, parallel controlled study	Kellgren/Lawrence grade 3–4 Aged 40–80 Patients between Aug ‘13- July ‘14	1 injection per group given once Anatomically guided injections 4 ml LP-PRP 2 ml betamethasone	6 months	VAS, KOOS and SF36 scores at 1, 3- and 6-months post-intervention

The Western Ontario and McMaster Universities Osteoarthritis Index (WOMAC) was the most frequently used outcome score (5 of 8 studies), with 2 of 8 using the Knee Injury and Osteoarthritis Outcome Score (KOOS) and 7 of 8 the visual analog scale (VAS). Khan et al. [29] reported WOMAC using individual sub-scores but did not calculate total scores. One study Freire et al. [28] reported results using the Knee Society Score (KSS) and Jubert et al. [22] additionally reported the SF-36 sub-score. Four studies reported outcomes at 1 month, 2 at 2 months, 5 at 3 months, 7 at 6 months, 1 at 9 months and 2 at 12 months.

Six of the eight individual studies reported that PRP was significantly better than CS, with the benefit most pronounced at 6 months, with two studies showing no significant difference between groups. Three studies used multiple PRP injections whereas six used single injections Uslu et al. [20] used both) and five studies used LR-PRP compared to three using LP-PRP (Huang et al. [26], Freire et al. [28], Jubert et al. [22]). No studies included patients with knee co-morbidities, knee injections within the previous 3 months or systemic diseases affecting the joints or blood (e.g., septicaemia, rheumatoid arthritis). Three of the studies were conducted in Europe, one in South America and four in Asia. Three studies recruited participants by approaching those attending their unit over a certain timeframe, one by asking for volunteers in the eligible patient population and one recruited through a list of patients referred to pain clinic. Three studies did not specify how patients were recruited.

### Preparation and dosages of PRP and corticosteroids

Whilst most studies used similar dosages and preparations of CS, there was considerable variability in the preparation of PRP, summarised in Additional file 3.

### Risk of Bias within studies

A summary of the results of the risk of bias assessment are presented in Additional file 4 using the Cochrane RoB2 tool [32]. Seven studies achieved a low risk of bias, whilst one study Camurcu et al. [31] was presumed at high risk of bias as no form of randomisation was implemented. It is worth noting that this tool does not assess blinding within its bias domains, and a more complete assessment with a justification of the evaluation for each domain is presented in Additional file 5. Additionally, not all of the studies blinded participants and/or physicians, however this was either deemed unethical or unsuitable by several of the study authors. Results suggest overall bias is low and is unlikely to affect the outcome of this review.

In four studies (50%), all patients were analysed, with no study drop-outs. Of the remaining four, mean attrition rate in the CS group was 11% (9–14%) and 2.25% (0–14%) in the PRP group. Reasons for attrition were justified in each paper. Adverse events were reported in four (50%) papers, and all were deemed to be mild and self-limiting.

### Sponsorship, publication Bias and heterogeneity of included studies

No paper appeared subject to sponsorship bias and we found no evidence of publication bias, for example, through searching the unpublished literature and journal abstracts for contradictory results. Considerable statistical heterogeneity was found across multiple timeframes; however, this was to be expected as many of the studies assessed different OA severity, across different timeframes, with different patient ages and PRP preparations. Moreover, similar studies assessing PRP compared with hyaluronic acid (HA) or HA compared to CS report comparable study heterogeneity [17, 33, 34].

### Outcomes by timeframe

**Fig. 2:**
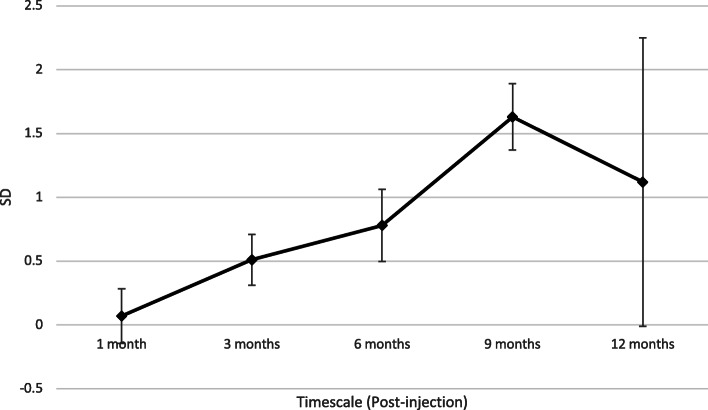
Temporal Relationship of the Additional Benefit of PRP (SD)

Our primary analysis was at 6 months follow up, where there was evidence that mean score in the PRP group was 0.78 SMD (*P < 0.005*) lower than in the CS group ((95% CI 0.12, 0.90)). Table 2 shows the results from each performed analysis. The forest plot these results are derived from is included as Additional file 6. On individual analysis of the WOMAC or VAS scales, this is represented by an additional reduction of 9.51 or 0.97 respectively. At 1 month of follow-up there was a non-significant additional reduction in overall symptoms in the PRP group compared to the CS group. At 3 months, six studies reported outcomes with a small but significant reduction (*P = 0.01*) in favour of the PRP group (− 0.51 SD). One study reported outcomes at 9 months (WOMAC only), with an additional reduction of 8.04 in the PRP group. At 12 months there was a statistically non-significant reduction in favour of the PRP group. These analyses are represented on their individual scales in Table 3. The temporal relationship of the additional benefit from PRP over CS injections is represented in Fig. 2.

**Table 2:** Results from each Performed Analysis (SMD)

Pain Score Reduction (WOMAC/VAS)					
Outcome	Number of studies	Pooled SMD (95 CI)	*P*	Heterogeneity	
				I^2^ (%)	*P*
1 month	4	−0.07 [−0.49, 0.35]	0.74	65	0.03
3 month	6	−0.51 [− 0.90, − 0.12]	**0.01**	72	0.003
6 month	7	−0.78 [− 1.34, − 0.23]	**0.005**	88	< 0.00001
9 months	1	− 1.63 [− 2.14, − 1.12]	**< 0.00001**	n/a	n/a
12 months	2	−1.12 [− 3.34, 1.09]	0.32	97	< 0.00001
LP-PRP	3	−0.61 [− 0.92, − 0.29]	**0.00002**	16	0.31
LR-PRP	5	−0.98 [−1.79, − 0.17]	**0.02**	92	< 0.00001
Single PRP injection	5	−0.52 [−1.11, 0.07]	**0.08**	87	< 0.00001
Triple PRP Injection	2^a^	−0.94 [−1.31, − 0.58]	**< 0.00001**	12	0.29

^a^Uslu et al. [20] excluded from the analysis for significant statistical heterogeneity

**Table 3:** Results from each Performed Analysis on both the WOMAC and VAS Scales

Outcome	Pooled mean difference (MD)					
	No. Studies	WOMAC (/96)	*P*	No. Studies	VAS (/10)	*P*
3 months	3	−2.15 [−5.65, 1.35]	0.23	4	− 0.51 [− 1.02, 0.01]	0.05
6 months	4	−9.51 [− 15.20, −3.83]	0.05	4	− 0.97 [− 1.94, 0.00]	0.05
6 months: KL 1–2	1	−3.86 [−6.01, − 1.71]	0.0004	1	−0.28 [− 0.97, 0.41]	0.43
6 months: KL 2–3	2	− 9.10 [− 15.73, − 2.46]	0.007	2	− 0.68 [− 2.01, 0.65]	0.32
9 months*	1	−8.04 [− 10.19, − 5.89]	< 0.00001	–	–	–
12 months	2	− 8.10 [− 23.86, 7.65]	0.31	2	−0.07 [− 0.42, 0.28]	0.69
LP-PRP	2	− 7.95 [− 18.14, 2.24]	0.13	2	−0.39 [− 1.01, 0.23]	0.22
LR-PRP	2	− 11.26 [− 19.88, − 2.64]	0.01	5	− 0.98 [− 1.80, − 0.16]	0.02
Triple PRP injection	2	−9.65 [− 21.35, 2.04]	0.11	3	−1.30 [− 2.33, − 0.26]	0.01
Single PRP Injection	2	−9.10 [− 15.75, − 2.46]	0.007	44	− 0.26 [− 1.03, 0.52]	0.52

### KL grade: outcomes at six months

**Fig. 3:**
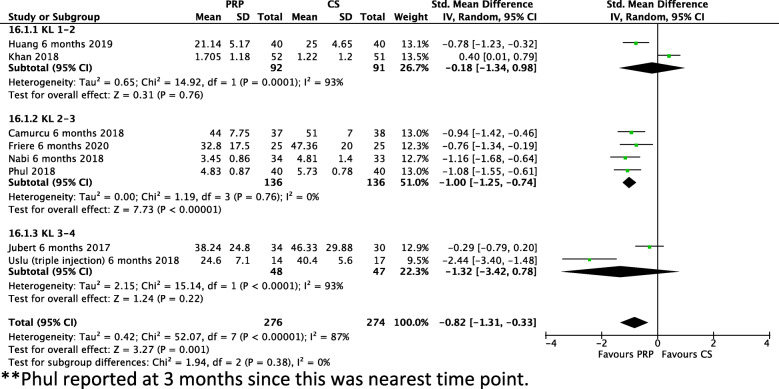
SD at 6 months Stratified by Kellegren-Lawrence Grade

As outcomes were most commonly reported at 6 months, these were stratified by KL grade to identify the efficacy of PRP across different severities of knee OA (Fig. 3). In KL 1–2, two studies reported a non-significant additional benefit for PRP (− 0.18 SMD, − 3.86 on WOMAC scale, − 0.28 on VAS) as did two studies in KL 3–4 (− 1.32 SMD, − 0.32 on VAS). In four studies including patients with KL 2–3 knee OA, a significant reduction (*P* < 0.00001) of 1 SMD was observed (I^2^ = 0%). On analysis of the individual scales this is represented as additional reduction of 9.1 on the WOMAC scale or 0.68 on the VAS scale.

### Subscore analysis

#### WOMAC subscores

Three studies reported WOMAC subscale scores at 6 months post-intervention. These results are displayed in Table 4**.** Statistically significant reductions in favour of PRP were observed in the WOMAC pain and stiffness subscales. A statistically non-significant reduction was observed in the physical function scale.

**Table 4:** WOMAC Subscale Scores at 6 Months Post-Intervention

WOMAC Subscale Score Reduction				
Outcome	Pooled mean difference (95 CI)	*P*	Heterogeneity	
			I^2^ (%)	*P*
Pain	−2.66 [−4.73, −0.60]	0.01	93	< 0.00001
Stiffness^a^	−1.95 [−2.30, − 1.59]	< 0.00001	0	0.78
Physical Function	−5.47 [− 13.80, 2.86]	0.2	97	0.00001

^a^Khan et al. (2018) [29] was removed from the analysis due to contributing 91% statistical heterogeneity

### KOOS sports/recreation scores

**Fig. 4:**
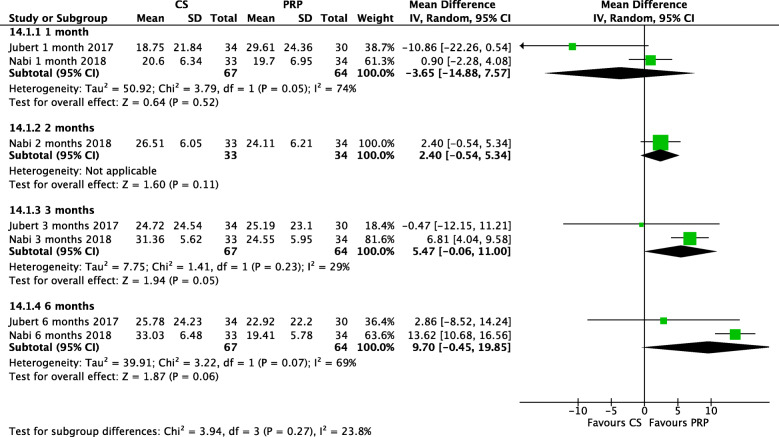
MD in KOOS Sports Score across Multiple Timeframes

As the WOMAC score does not specifically address sports outcomes, the KOOS sports/activities subscale (reported in two studies) was analysed (Fig. 4). At 1 month, CS and PRP injections were comparable (*P* = 0.52, magnitude 3.65). After this point, PRP was favoured, being statistically significant at 3 months (*P* = 0.05), with an improvement of 5.7 (I^2^ = 29%). At 6 months, a near significant (*P* = 0.06) improvement was observed in favour of PRP, of magnitude 9.7.

### LP-PRP versus LR-PRP

Both LP-PRP and LR-PRP significantly reduced scores when compared with CS. LP-PRP showed an additional improvement of 0.61 SMD compared to CS (*P* = 0.00002, I^2^ = 16%) whereas LR-PRP showed additional reduction of 0.98 SMD (*P* = 0.02, I^2^ = 92%); thus LR-PRP appears more effective in reducing pain than LP-PRP. The reduction in WOMAC and VAS scores as a result is demonstrated in Table 3. Although LR-PRP appears to have a greater effect in this analysis compared to LP-PRP, significant statistical heterogeneity may explain some of the difference between these groups and should be considered when interpreting our results.

### Single versus triple injections of PRP

Single injections showed an additional benefit over CS of 0.52 SMD (*P* = 0.08, I^2^ = 87%) and triple injections had an additional benefit of 0.94 SMD (*P* < 0.00001, I^2^ = 12%), suggesting three injections are superior to one. It is worth noting that Uslu et al. [20] did not perform three CS injections in the control group, which may affect results; Uslu et al. [20] was further excluded from the analysis due to significant statistical heterogeneity, but found superior results with three injections of PRP. Both Khan et al. [29] and Naderi et al. [30] matched the number of injections given in the control groups, and these were spaced at equal timeframes.

## Discussion

This is the first systematic review comparing IA-PRP to IA-CS injections for the symptomatic management of knee OA and our meta-analysis shows that IA-PRP shows greater overall efficacy compared to IA-CS injections for knee OA over 12 months of follow-up. This effect becomes statistically significant from 3 months onwards, and is most pronounced from 6 to 9 months, with the strongest evidence of benefit at 6 months post-injection. The benefits appear to be greatest in patients with mild to moderate knee OA (KL 2–3). PRP shows greater efficacy in reducing pain scores whilst improving stiffness and general physical function, as well as participation in sports or other activities, when compared to CS injections. Three injections of PRP showed a significantly greater effect than one injection, whilst there is a suggestion that LR-PRP is more effective than LP-PRP. Thus, the take home message for practitioners is that one to three IA injections of LR-PRP should now be offered for symptomatic management of mild-moderate knee OA (KL grade 2–3 on knee x-ray) as opposed to IA-CS injections and could be repeated on a six-monthly basis.

### Efficacy by timeframe

With reference to CS injections, our results are in keeping with the literature, in that they appear equally efficacious to PRP injections at 1 month post-injection. A Cochrane review on the use of CS injections for knee OA conclude that the effects of the injection decreases over time, with no evidence to suggest that an effect remains 6 months post-injection [12]. Similarly, both this review and another review of Level 1 studies found that the effect from IA CS injections were only significant at 1 week [12, 35], with a general consensus that there is little benefit from the injections after a period of 6 weeks [35, 36].

Pain and stiffness scores in the PRP group continue to improve until 9 months post-injection (Fig. 2), with statistical significance from three months onwards. This is supported by previous evidence by Shen et al. [34] and Filardo et al. [37], who suggested a sustained effect following PRP injections of up to 12 and even 24 months [34, 37]. The former study was a systematic review and meta-analysis investigating the temporal effect of PRP injections compared to control (including HA, CS, placebo and ozone) which reported superior treatment effects from PRP at 3, 6 and 12 months; the latter was a single arm study investigating whether beneficial effects from PRP persist. At 24 months, patient scores were still improved compared to baseline, but the median duration of effect was 9 months. Our systematic review and meta-analysis showed that 6–9 months was the time point when patients will observe the greatest treatment effect from IA-PRP injections.

Seven of the eight studies reported results at 6 months post-injection; as such, results could be stratified by KL grade. The greatest significant effect was reported in KL 2–3 (early-moderate OA), supported by an earlier study finding that early OA showed a greater response and duration of effect than in severe OA [38]. In a review by Meheux et al. [17] comparing PRP to HA and placebo for knee OA, PRP was recommended to be reserved for patients with KL 1–3 [17]. This may be due to the ability of PRP to restore and protect cartilage [39, 40], as this may be more effective where there is more pre-existing joint cartilage.

### Magnitude of improvement

A Cochrane review comparing CS to placebo injections reported a reduction of 0.4 SMD, or 1 on the VAS pain scale, compared to our observed additional mean reduction in the PRP group of − 0.51 SMD at 3 months or − 0.78 SMD at 6 months. In context, our results suggest the effect from PRP is nearly double that again of CS injections [12]. A recent systematic review comparing PRP to HA found that PRP produced an additional reduction of − 20.69 on the WOMAC scale at 6 months, with our results showing a reduction of − 9.51 compared to CS at the same timeframe [41]. Indeed the minimally clinically important difference (MCID) in knee OA has been previously identified as an improvement on the WOMAC scale of 1.33 points [42], of which these results are well in excess of at 6 months of follow-up. Patients should be reassured that their symptoms will continue to improve in the months post-injection, receiving the greatest relief as they approach six months.

### Subscore analysis: pain, stiffness and sporting participation

On WOMAC subscore analysis, at 6 months pain and stiffness were significantly improved in the PRP group compared to CS, but overall function was not. This would suggest that most of the perceived benefit from PRP at this timeframe is symptomatic, and this hypothesis is reinforced by Filardo et al. [37]. The greatest effect in sporting improvement on the KOOS subscale was also observed at 6 months, likely due to reduced pain and stiffness improving sporting participation; physical activity often being a co-prescription which may in itself help improve OA symptoms [43, 44]. An improvement in sporting participation and exercise is additionally desirable in promoting weight loss, which can have a profound effect on improving function in knee OA [45].

### Leukocyte-rich versus leukocyte-poor PRP

We found a slightly greater effect from LR- compared with LP-PRP injections, however this was not significant between groups. LR-PRP contains more pro-inflammatory mediators whereas LP-PRP contains less, and there is debate over which is of greater benefit [21]. On one hand, it has been suggested that an initial pro-inflammatory phase is important for regeneration [46], whilst on the other hand, a greater concentration of anti-inflammatory mediators in LP-PRP is presumed favourable [47]. Our results advocate the former hypothesis, but this requires further investigation.

### Number of PRP injections

Another topic of debate, previously identified [48], is whether single or multiple PRP injections provide different treatment effects. We demonstrated a greater treatment effect with the use of multiple injections. Previous research has found either no benefit or harm from multiple injections [49] or that multiple injections improve joint functionality [50], with the caveat that multiple injection sites may increase risk of local reaction [48]. Adding to this, a recent RCT comparing PRP to HA injections found that triple injections were significantly better than single injections, but only in low grade OA [21]. A Guinea pig model found that both short term and long term benefits were more sustained and pronounced in early grade knee OA [51], and that only multiple injections stimulated a chondroprotective effect, suggesting a threshold dosage and that multiple injections improve the duration of effect of PRP. Thus PRP injections, both single and multiple, appear safe to use, with multiple injections appearing to offer greater symptomatic improvement than single PRP injections.

### OA grade and age and response to treatment

It has been proposed that outcomes following IA PRP injections are generally best for mild-moderate grade OA, and our results are in keeping with this (KL 2–3) [37, 48]. Moreover, it has been suggested that PRP injection efficacy has a correlation with age [37], PRP showing a greater efficacy in those under 50 [52]. This was, however, not something we could analyse.

### Adverse effects

Although adverse events were only reported in four (50%) of the included studies, no serious complications were recorded. Previous evidence indicates that all adverse effects tend to be non-specific, mild and self-limiting [34, 38, 53]. No joint infections were reported in the follow-up period of the included studies.

### Strengths

The strengths of this review include analysing only prospective trials using evidence-based medicine techniques and taking a systematic approach to study analysis using validated quality assessment tools. We previously submitted our protocol paper for publication (awaiting outcome of peer review) and all included studies had adequate follow up (often with multiple timeframes), with a low attrition rate, low risk of bias and relatively homogenous populations. The use of standard, validated outcome measurements (WOMAC, VAS and KOOS) across studies is ideal for direct comparison.

### Limitations

Studies varied in the lengths of time that patients had OA, as well as patient age. There were considerable differences in sex distribution between studies, with more females being included. High statistical heterogeneity among studies was also a noted limitation; however, similar reviews on PRP injections for knee OA report a similar observation [17, 33, 34]. This could be for several reasons. The preparation, centrifugation, leukocyte concentration and dosage of PRP may have considerable effect on its efficacy [54–56], and even within the same patients, across multiple blood draws, the PRP yield may vary [57]. Methods of measuring platelet count also effect reporting accuracy [58]. Differences in the preparation of PRP has been shown to directly implicate its chondro- and osteo- inductive properties in vitro [59]. A systematic review by Chahla et al. [60] investigating PRP preparation protocols and composition found that only 10% of studies included a description of PRP preparation and 16% on its composition, concluding that this is sub-optimal for the comparison of PRP products and advocating for detailed and stepwise descriptions in all further publications. This is an area where further research and ultimately the development of standardised procedures are needed, with both Dhurat and Sukesh [55] and Chahla et al. [60] reporting advice and suggestions for what should be included in a standardised PRP protocol.

## Conclusions

This systematic review and meta-analysis indicates that IA-PRP produces favorable outcomes when compared with CS injections in the management of knee OA, including improved pain management, less joint stiffness and better participation in exercise/physical activity, including sports. This symptomatic improvement lasts for at least 6 months and giving three IA-PRP, with injections typically separated by a week, appears more effective than one IA-PRP injection. There is limited information in this review comparing LR−/LP- PRP, with LR-PRP appearing more effective than LP-PRP for symptomatic management of knee OA, but this requires further investigation. Further studies into the effectiveness of IA-PRP injections should standardise the preparation and content of the injectate as well as report a detailed PRP preparation protocol.

## Supplementary Information

**Additional file 1.** Search Strategy.

**Additional file 2.** Example Funnel Plot from Primary Analysis.

**Additional file 3.** Preparation and Dosage of PRP and CS injectate.

**Additional file 4.** Summary of Risk of Bias in Included Studies.

**Additional file 5.** Risk of Bias Assessment.

**Additional file 6.** Forest plot of Main Analyses Performed (SMD).

## Data Availability

All data generated or analysed during this study are included in this published article [and its supplementary information files].

## Abbreviations

IA: Intra-articular
CS: Corticosteroid(s)
OA: Osteoarthritis
PRP: Platelet-Rich Plasma
MS: Musculoskeletal
GP: General Practice
ACL: Anterior Cruciate Ligament
UK: United Kingdom
LR-PRP: Leukocyte-Rich PRP
LP-PRP: Leukocyte-Poor PRP
PRISMA: Preferred Reporting Items for Systematic Reviews and Meta-Analyses
PROSPERO: The International Prospective Register of Systematic Reviews
WOMAC: Western Ontario and McMaster Universities Osteoarthritis Index
KOOS: Knee Injury and Osteoarthritis Outcome Score
VAS: Visual Analogue Scale
KL: Kellgren Lawrence
SE: Standard Error
SMD: Standardised mean difference MD Mean difference
BMI: Body Mass Index
SF-36: Short Form (36) Health Survey
MCID: Minimally Clinically Important Difference
HA: Hyaluronic Acid

## References

[1] Jordan KP, Kadam UT, Hayward R, Porcheret M, Young C, Croft P (2010). Annual consultation prevalence of regional musculoskeletal problems in primary care: an observational study. BMC Musculoskelet Disord.

[2] Boshuizen HC, Poos MJJC, van den Akker M, van Boven K, Korevaar JC, de Waal MWM, Biermans MCJ, Hoeymans N (2017). Estimating incidence and prevalence rates of chronic diseases using disease modeling. Popul Health Metrics.

[3] Heron N, Ryans I. What Musculoskeletal (MSK) Conditions are Referred from Routine General Practice (GP) and what Impact does this have on Developing Innovative Care Models for Patients with MSK Conditions in Primary Care? Int J Phys Med Rehabil. 2016;4(06).

[4] Cross M, Smith E, Hoy D, Nolte S, Ackerman I, Fransen M, Bridgett L, Williams S, Guillemin F, Hill CL, Laslett LL, Jones G, Cicuttini F, Osborne R, Vos T, Buchbinder R, Woolf A, March L (2014). The global burden of hip and knee osteoarthritis: estimates from the global burden of disease 2010 study. Ann Rheum Dis.

[5] GBD2017 Disease and Injury Incidence and Prevalence Collaborators. Global, regional, and national incidence, prevalence, and years lived with disability for 354 diseases and injuries for 195 countries and territories, 1990–2017: a systematic analysis for the Global Burden of Disease Study 2017. Lancet. 2018;392(10159):1789–858. 10.1016/S0140-6736(18)32279-7 PMC6227754 30496104

[6] Felson DT, Lawrence RC, Dieppe PA, Hirsch R, Helmick CG, Jordan JM, Kington RS, Lane NE, Nevitt MC, Zhang Y, Sowers M, McAlindon T, Spector TD, Poole AR, Yanovski SZ, Ateshian G, Sharma L, Buckwalter JA, Brandt KD, Fries JF (2000). Osteoarthritis: new insights. Part 1: the disease and its risk factors. Ann Intern Med.

[7] National CGCU (2014). Osteoarthritis: care and management in adults.

[8] Wallis JA, Webster KE, Levinger P, Taylor NF (2013). What proportion of people with hip and knee osteoarthritis meet physical activity guidelines? A systematic review and meta-analysis. Osteoarthr Cartil.

[9] Harrison A, Appleby J. The war on waiting for hospital treatment: what has labour achieved and what challenges remain: summary: King's Fund; 2005.

[10] Appleby J (2019). Waiting times compared across the four UK nations. BMJ..

[11] Morris J, Twizeyemariya A, Grimmer K (2018). What is the current evidence of the impact on quality of life whilst waiting for management/treatment of orthopaedic/musculoskeletal complaints? A systematic scoping review. Qual Life Res.

[12] Juni P, Hari R, Rutjes AW, Fischer R, Silletta MG, Reichenbach S, et al. Intra-articular corticosteroid for knee osteoarthritis. Cochrane Database Syst Rev. 2015;10(10):1–80. https://www.cochranelibrary.com/cdsr/doi/10.1002/14651858.CD005328.pub3/epdf/full. 10.1002/14651858.CD005328.pub3 PMC8884338 26490760

[13] McAlindon TE, LaValley MP, Harvey WF, Price LL, Driban JB, Zhang M (2017). Effect of intra-articular triamcinolone vs saline on knee cartilage volume and pain in patients with knee osteoarthritis: a randomized clinical trial. JAMA..

[14] Dragoo JL, Danial CM, Braun HJ, Pouliot MA, Kim HJ (2012). The chondrotoxicity of single-dose corticosteroids. Knee Surg Sports Traumatol Arthrosc.

[15] Zeng C, Lane NE, Hunter DJ, Wei J, Choi HK, McAlindon TE (2019). Intra-articular corticosteroids and the risk of knee osteoarthritis progression: results from the osteoarthritis initiative. Osteoarthr Cartil.

[16] Dai WL, Zhou AG, Zhang H, Zhang J (2017). Efficacy of Platelet-Rich Plasma in the Treatment of Knee Osteoarthritis: A Meta-analysis of Randomized Controlled Trials. Arthroscopy.

[17] Meheux CJ, McCulloch PC, Lintner DM, Varner KE, Harris JD (2016). Efficacy of intra-articular platelet-rich plasma injections in knee osteoarthritis: a systematic review. Arthroscopy.

[18] Kabiri A, Esfandiari E, Esmaeili A, Hashemibeni B, Pourazar A, Mardani M (2014). Platelet-rich plasma application in chondrogenesis. Adv Biomed Res.

[19] Sundman EA, Cole BJ, Karas V, Della Valle C, Tetreault MW, Mohammed HO, Fortier LA (2014). The anti-inflammatory and matrix restorative mechanisms of platelet-rich plasma in osteoarthritis. Am J Sports Med.

[20] Uslu Guvendi E, Askin A, Guvendi G, Kocyigit H (2018). Comparison of efficiency between corticosteroid and platelet rich plasma injection therapies in patients with knee osteoarthritis. Arch Rheumatol.

[21] Görmeli G, Görmeli CA, Ataoglu B, Çolak C, Aslantürk O, Ertem K (2017). Multiple PRP injections are more effective than single injections and hyaluronic acid in knees with early osteoarthritis: a randomized, double-blind, placebo-controlled trial. Knee Surg Sports Traumatol Arthrosc.

[22] Joshi Jubert N, Rodríguez L, Reverté-Vinaixa MM, Navarro A (2017). Platelet-Rich Plasma Injections for Advanced Knee Osteoarthritis: A Prospective, Randomized, Double-Blinded Clinical Trial. Orthop J Sports Med.

[23] Riboh JC, Saltzman BM, Yanke AB, Fortier L, Cole BJ (2016). Effect of leukocyte concentration on the efficacy of platelet-rich plasma in the treatment of knee osteoarthritis. Am J Sports Med.

[24] Moher D, Liberati A, Tetzlaff J, Altman DG, The PG (2009). Preferred reporting items for systematic reviews and meta-analyses: The PRISMA statement. PLoS Med.

[25] Hozo SP, Djulbegovic B, Hozo I (2005). Estimating the mean and variance from the median, range, and the size of a sample. BMC Med Res Methodol.

[26] Huang Y, Liu X, Xu X, Liu J. Intra-articular injections of platelet-rich plasma, hyaluronic acid or corticosteroids for knee osteoarthritis: A prospective randomized controlled study. Orthopade. 2019;48(3):239–47. 10.1007/s00132-018-03659-5 30623236

[27] Phul SH, Mobushir M, Jilani RUA, Khan IS, Malik RH, Jan G. Comparison of Intra-Articular Steroids Injection Versus Platelets Rich Plasma Injection in Patients with Osteoarthritic Knee Joints; 2018.

[28] Freire MRdM, da Silva PMC, Azevedo AR, Silva DS, da Silva RBB, Cardoso JC. Comparative effect between infiltration of platelet-rich plasma and the use of corticosteroids in the treatment of knee osteoarthritis: a prospective and randomized clinical trial. Revista Brasileira de Ortopedia. 2020;55(5):551–6. 10.1016/j.rbo.2018.01.001 PMC7575359 33093718

[29] KHAN AF, GILLANI SFUHS, Khan AF. Role of Intra-Articular Corticosteroid with Xylocaine Vs Plate Rich Plasma for the Treatment of Early Grade II Knee Osteoarthritis at Akhtar Saeed Teaching Hospital Lahore: A Randomized Controlled Trail. 2018.

[30] Naderi NB, Sedighinejad A, Mardani-Kivi M, Haghighi M, Atrkar Roushan Z, Ghazanfar Tehran S, Biazar G. Comparing the effectiveness of intraarticular platelet-rich plasma and corticosteroid injection under ultrasound guidance on pain control of knee osteoarthritis. Iranian Red Crescent M J. 2018;20(3).

[31] Camurcu Y, Sofu H, Ucpunar H, Kockara N, Cobden A, Duman S: Single-dose intra-articular corticosteroid injection prior to platelet-rich plasma injection resulted in better clinical outcomes in patients with knee osteoarthritis: A pilot study. J Back Musculoskeletal Rehabilitation. 2018;31(4):603–10. 10.3233/BMR-171066 29710676

[32] McGuinness L, Higgins J. Risk-of-Bias VISualization (robvis): an R package and shiny web app for visualizing risk-of-bias assessments. Res Synth Methods. 2020. 10.1002/jrsm.1411 32336025

[33] Chang KV, Hung CY, Aliwarga F, Wang TG, Han DS, Chen WS (2014). Comparative effectiveness of platelet-rich plasma injections for treating knee joint cartilage degenerative pathology: a systematic review and meta-analysis. Arch Phys Med Rehabil.

[34] Shen L, Yuan T, Chen S, Xie X, Zhang C (2017). The temporal effect of platelet-rich plasma on pain and physical function in the treatment of knee osteoarthritis: systematic review and meta-analysis of randomized controlled trials. J Orthop Surg Res.

[35] Hepper CT, Halvorson JJ, Duncan ST, Gregory AJM, Dunn WR, Spindler KP (2009). The Efficacy and Duration of IntrÅ articular Corticosteroid Injection for Knee Osteoarthritis: A Systematic Review of Level I Studies. J Am Acad Orthop Surg.

[36] Orchard JW (2020). Corticosteroid injections: glass half-full, half-empty or full then empty?. Br J Sports Med.

[37] Filardo G, Kon E, Buda R, Timoncini A, Di Martino A, Cenacchi A (2011). Platelet-rich plasma intra-articular knee injections for the treatment of degenerative cartilage lesions and osteoarthritis. Knee Surg Sports Traumatol Arthrosc.

[38] Jang SJ, Kim JD, Cha SS (2013). Platelet-rich plasma (PRP) injections as an effective treatment for early osteoarthritis. Eur J Orthop Surg Traumatol.

[39] Liu J, Song W, Yuan T, Xu Z, Jia W, Zhang C (2014). A comparison between platelet-rich plasma (PRP) and hyaluronate acid on the healing of cartilage defects. PLoS One.

[40] Kon E, Buda R, Filardo G, Di Martino A, Timoncini A, Cenacchi A (2010). Platelet-rich plasma: intra-articular knee injections produced favorable results on degenerative cartilage lesions. Knee Surg Sports Traumatol Arthrosc.

[41] Wu Q, Luo X, Xiong Y, Liu G, Wang J, Chen X, Mi B (2020). Platelet-rich plasma versus hyaluronic acid in knee osteoarthritis: a meta-analysis with the consistent ratio of injection. J Orthop Surg.

[42] Angst F, Aeschlimann A, Stucki G. Smallest detectable and minimal clinically important differences of rehabilitation intervention with their implications for required sample sizes using WOMAC and SF-36 quality of life measurement instruments in patients with osteoarthritis of the lower extremities. Arthritis Care Res. 2001;45(4):384–91. 10.1002/1529-0131(200108)45:4<384::AID-ART352>3.0.CO;2-0. 10.1002/1529-0131(200108)45:4<384::AID-ART352>3.0.CO;2-0 11501727

[43] Schulz JM, Birmingham TB, Atkinson HF, Woehrle E, Primeau CA, Lukacs MJ, al-Khazraji BK, Khan MCM, Zomar BO, Petrella RJ, Beier F, Appleton CT, Shoemaker JK, Bryant DM (2020). Are we missing the target? Are we aiming too low? What are the aerobic exercise prescriptions and their effects on markers of cardiovascular health and systemic inflammation in patients with knee osteoarthritis? A systematic review and meta-analysis. Br J Sports Med.

[44] Guidelines ACoRSoO. Recommendations for the medical management of osteoarthritis of the hip and knee: 2000 update. Arthritis Rheum. 2000;43(9):1905–15. 10.1002/1529-0131(200009)43:9<1905::AID-ANR1>3.0.CO;2-P. 10.1002/1529-0131(200009)43:9<1905::AID-ANR1>3.0.CO;2-P 11014340

[45] Christensen R, Astrup A, Bliddal H (2005). Weight loss: the treatment of choice for knee osteoarthritis? A randomized trial. Osteoarthr Cartil.

[46] Lana JF, Huber SC, Purita J, Tambeli CH, Santos GS, Paulus C, Annichino-Bizzacchi JM (2019). Leukocyte-rich PRP versus leukocyte-poor PRP - The role of monocyte/macrophage function in the healing cascade. J Clin Orthop Trauma.

[47] Le ADK, Enweze L, DeBaun MR, Dragoo JL (2018). Current clinical recommendations for use of platelet-rich plasma. Curr Rev Musculoskelet Med.

[48] Campbell KA, Saltzman BM, Mascarenhas R, Khair MM, Verma NN, Bach BR, Cole BJ (2015). Does intra-articular platelet-rich plasma injection provide clinically superior outcomes compared with other therapies in the treatment of knee osteoarthritis? A systematic review of overlapping meta-analyses. Arthroscopy..

[49] Orscelik A, Yildiz Y (2015). Comparison of single and triple platelet rich plasma injections in the treatment of Patellofemoral pain syndrome. Turk Klin J Med Sci.

[50] Vilchez-Cavazos F, Millán-Alanís JM, Blázquez-Saldaña J, Álvarez-Villalobos N, Peña-Martínez VM, Acosta-Olivo CA (2019). Comparison of the Clinical Effectiveness of Single Versus Multiple Injections of Platelet-Rich Plasma in the Treatment of Knee Osteoarthritis: A Systematic Review and Meta-analysis. Orthop J Sports Med.

[51] Chouhan DK, Dhillon MS, Patel S, Bansal T, Bhatia A, Kanwat H (2019). Multiple platelet-rich plasma injections versus single platelet-rich plasma injection in early osteoarthritis of the knee: an experimental study in a Guinea pig model of early knee osteoarthritis. Am J Sports Med.

[52] Kon E, Mandelbaum B, Buda R, Filardo G, Delcogliano M, Timoncini A, Fornasari PM, Giannini S, Marcacci M (2011). Platelet-rich plasma intra-articular injection versus hyaluronic acid Viscosupplementation as treatments for cartilage pathology: from early degeneration to osteoarthritis. Arthroscopy.

[53] Rayegani SM, Raeissadat SA, Taheri MS, Babaee M, Bahrami MH, Eliaspour D (2014). Does intra articular platelet rich plasma injection improve function, pain and quality of life in patients with osteoarthritis of the knee? A randomized clinical trial. Orthop Rev (Pavia).

[54] Everts PAM, Brown Mahoney C, Hoffmann JJML, Schönberger JPAM, Box HAM, van Zundert A, Knape JTA (2006). Platelet-rich plasma preparation using three devices: implications for platelet activation and platelet growth factor release. Growth Factors.

[55] Dhurat R, Sukesh M (2014). Principles and methods of preparation of platelet-rich plasma: a review and Author's perspective. J Cutan Aesthet Surg.

[56] Laver L, Marom N, Dnyanesh L, Mei-Dan O, Espregueira-Mendes J, Gobbi A (2017). PRP for degenerative cartilage disease: a systematic review of clinical studies. Cartilage..

[57] Mazzocca AD, McCarthy MBR, Chowaniec DM, Cote MP, Romeo AA, Bradley JP (2012). Platelet-rich plasma differs according to preparation method and human variability. JBJS..

[58] Mani H, Luxembourg B, Kläffling C, Erbe M, Lindhoff-Last E (2005). Use of native or platelet count adjusted platelet rich plasma for platelet aggregation measurements. J Clin Pathol.

[59] Han B, Woodell-May J, Ponticiello M, Yang Z, Nimni M (2009). The effect of thrombin activation of platelet-rich plasma on demineralized bone matrix osteoinductivity. J Bone Joint Surg Am.

[60] Chahla J, Cinque ME, Piuzzi NS, Mannava S, Geeslin AG, Murray IR, et al. A Call for Standardization in Platelet-Rich Plasma Preparation Protocols and Composition Reporting: A Systematic Review of the Clinical Orthopaedic Literature. JBJS. 2017;99(20). 10.2106/JBJS.16.01374 29040132

