# Supplementary material for: Intra-articular platelet-rich plasma injections versus intra-articular corticosteroid injections for symptomatic management of knee osteoarthritis: systematic review and meta-analysis
Source: BMC Musculoskelet Disord. 2021 Jun 16;22:550. doi: 10.1186/s12891-021-04308-3 (PMC8208610; doi:10.1186/s12891-021-04308-3)
Supplement: Supplementary file 1 — Additional file 1. Search Strategy. [file 12891_2021_4308_MOESM1_ESM.docx]

**Additional File 1: Search Strategy**

**Databases Searched:** MEDLINE, EMBASE, Web of Science, Scopus

**Sample Search Strategy:** TOPIC: (knee* near/3 osteoarthritic*) AND TOPIC: ("platelet rich plasma*")

**Timeline:** Inception – present (June 2020)
