# Supplementary material for: Intra-articular platelet-rich plasma injections versus intra-articular corticosteroid injections for symptomatic management of knee osteoarthritis: systematic review and meta-analysis
Source: BMC Musculoskelet Disord. 2021 Jun 16;22:550. doi: 10.1186/s12891-021-04308-3 (PMC8208610; doi:10.1186/s12891-021-04308-3)
Supplement: Supplementary file 2 — Additional file 2. Example Funnel Plot from Primary Analysis. [file 12891_2021_4308_MOESM2_ESM.docx]

**Additional File 2: Example Funnel Plot from Primary Analysis**
