# Supplementary material for: Intra-articular platelet-rich plasma injections versus intra-articular corticosteroid injections for symptomatic management of knee osteoarthritis: systematic review and meta-analysis
Source: BMC Musculoskelet Disord. 2021 Jun 16;22:550. doi: 10.1186/s12891-021-04308-3 (PMC8208610; doi:10.1186/s12891-021-04308-3)
Supplement: Supplementary file 3 — Additional file 3. Preparation and Dosage of PRP and CS injectate. [file 12891_2021_4308_MOESM3_ESM.docx]

**Additional File 3: Preparation and Dosage of PRP and CS injectate**

| **Study** | **How was injection given?** | **Steroid Used** | **Dosage** | **PRP Preparation** | **Dosage** |
| --- | --- | --- | --- | --- | --- |
| Huang et al. (2019) | Anatomically guided | Corticosteroid prepared by Shanghai Schering-Plough | 1mL | Single centrifugation, 5 min at 1500g | 4mL |
| Khan et al. (2018) | Anatomically guided | triamcinolone acetonide | 40mg | Preparation methods were not specified | 5mL |
| Nabi et al. (2018) | Ultrasound guided | Triamcinolone (NS) | 40mg | Double centrifugation, second at 2700rpm for 6 mins | 5mL |
| Friere et al. (2018) | Anatomically guided | triamcinolone acetate | 50mg | Double centrifugation as described by Sonnleitner et al | 5mL |
| Camurcu et al. (2018) | Anatomically guided | Methylprednisolone (not specified) | 1mL | Centrifugation at 1800 rpm for 8 mins | 3mL |
| Phul et al. (2018) | Anatomically guided | triamcinolone hexacetonide | 40mg | Double centrifugation, first with 1600rpm at 15 min, second 2800rpm for 7 mins | 4-6micromL |
| Uslu et al. (2017) | Anatomically guided | betamethasone | 7mg | Single centrifugation 5 min at 3600rpm | NOS |
| Jubert et al. (2017) | Anatomically guided | betamethasone | 6mg of both betamethasone sodium phosphate and betamethasone acetate | Double centrifugation, initially 280g for 15 mins then 680g for 20 minutes. Additional anticoagulant was added | 4mL |
