# Supplementary figures and images for: Intra-articular platelet-rich plasma injections versus intra-articular corticosteroid injections for symptomatic management of knee osteoarthritis: systematic review and meta-analysis
Source: BMC Musculoskelet Disord. 2021 Jun 16;22:550. doi: 10.1186/s12891-021-04308-3 (PMC8208610; doi:10.1186/s12891-021-04308-3)

**Additional File 4: Summary of Risk of Bias in Included Studies
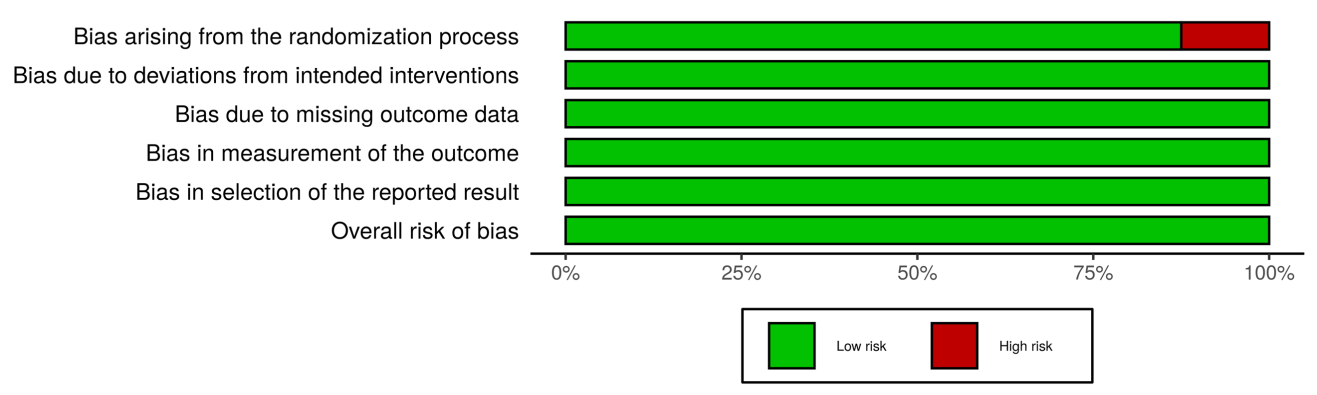
**

Supplement: Supplementary file 4 — Additional file 4. Summary of Risk of Bias in Included Studies. [file 12891_2021_4308_MOESM4_ESM.docx]

**Additional File 6: Forest plot of Main Analyses Performed (SMD)
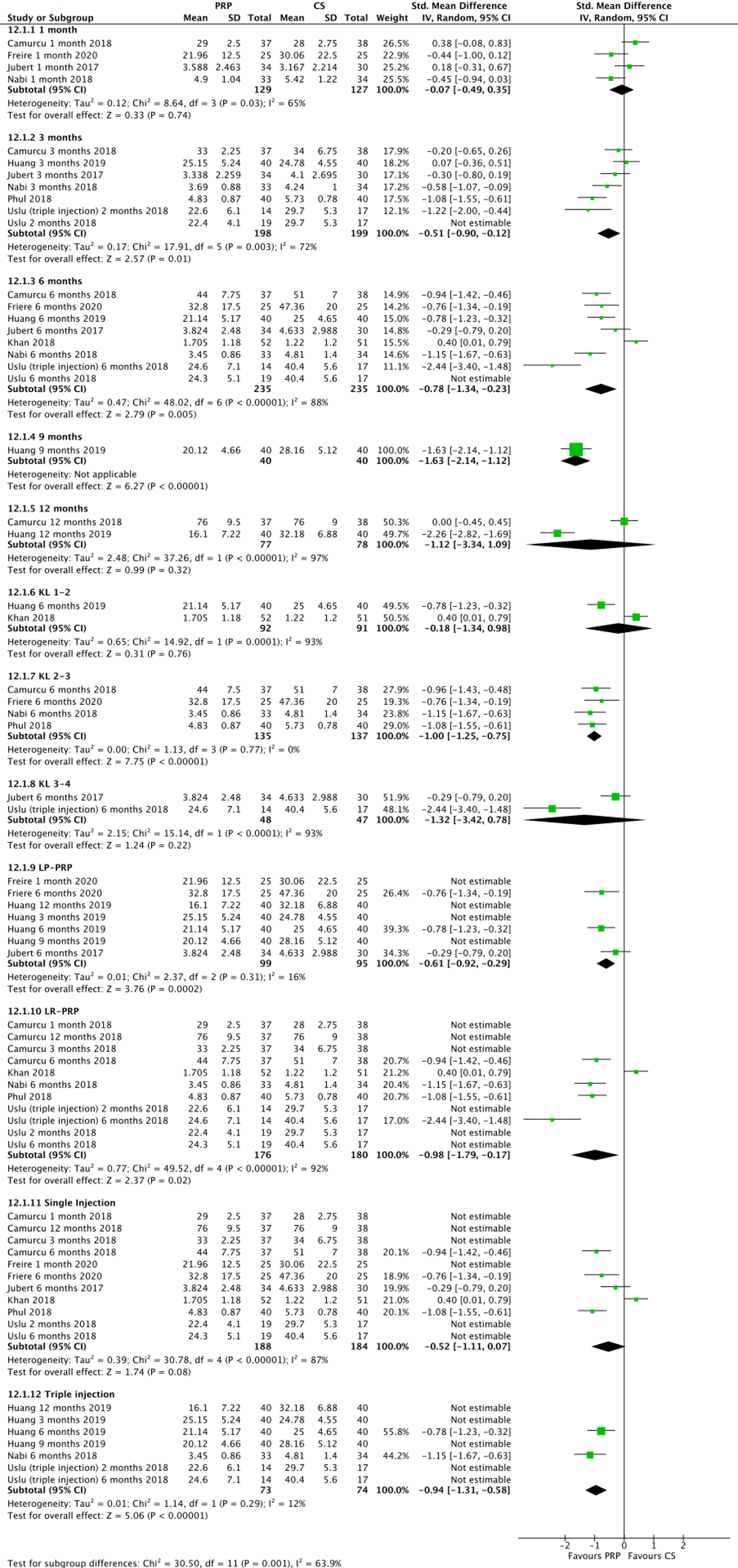
**

Supplement: Supplementary file 6 — Additional file 6. Forest plot of Main Analyses Performed (SMD). [file 12891_2021_4308_MOESM6_ESM.docx]
