# Supplementary material for: Intra-articular platelet-rich plasma injections versus intra-articular corticosteroid injections for symptomatic management of knee osteoarthritis: systematic review and meta-analysis
Source: BMC Musculoskelet Disord. 2021 Jun 16;22:550. doi: 10.1186/s12891-021-04308-3 (PMC8208610; doi:10.1186/s12891-021-04308-3)
Supplement: Supplementary file 5 — Additional file 5. Risk of Bias Assessment. [file 12891_2021_4308_MOESM5_ESM.docx]

**Additional File 5: Risk of Bias Assessment**

|  | Huang et al. (2019) | Khan et al. (2018) | Nabi et al. (2018) | Friere et al. (2018) | Camurcu et al. (2018) | Phul et al. (2018) | Uslu et al. (2017) | Jubert et al. (2017) |
| --- | --- | --- | --- | --- | --- | --- | --- | --- |
| **Random sequence generation (selection bias)** | Low risk | Low risk  Randomisation by lottery | Low risk  Quadruple block randomisation | Low risk  Block randomisation | High risk  No randomisation | Low risk | Low risk  Random allocation sequence | Low risk |
| **Allocation concealment (selection bias)** | Unclear risk | Low risk | Low risk | Low risk  Double blinding | High risk | Low risk | Low risk | Low risk  Double blinding |
| **Blinding of participants and researchers (performance bias)** | Low risk | Unclear risk | Low risk  Single (physician) blinded- deemed unethical to double blind and this is stated | Low risk | High risk | Unclear risk  No blinding | Low risk  Participants weren’t blinded, physicians were. | Low risk |
| **Blinding of outcome assessment (detection bias)** | Unclear risk  No blinding however standard survey/outcome tool used before and after | Unclear risk | Low risk | Low risk | Unclear risk | Unclear risk  No blinding, however unlikely to affect outcome | Low risk | Low risk |
| **Incomplete outcome data (attrition bias)** | Low risk  No patients lost to follow up, all patients excluded were justified | Low risk | Low risk | Low risk | Low risk | Low risk | Low risk | Low risk  Only 1 patient lost to follow-up |
| **Selective reporting (reporting bias)** | Low risk | Low risk | Low risk | Low risk | Low risk | Low risk | Low risk | Low risk |
| **Other bias** | Low risk | Low risk | Low risk | Low risk | Low risk | Low risk | Low risk | Low risk |
| **Overall Assessment** | Unclear risk | Unclear risk | Low risk | Low risk | High risk | Unclear risk | Low risk | Low risk |
